# Supplementary material for: Building better outcomes: A grounded theory approach to understanding creation and management of surgical data systems in Ethiopia
Source: PLOS Glob Public Health. 2026 Jan 30;6(1):e0005355. doi: 10.1371/journal.pgph.0005355 (PMC12857948; doi:10.1371/journal.pgph.0005355)
Supplement: S1 File — (DOCX) [file pgph.0005355.s001.docx]

**Objective of study:** Identify barriers and facilitators to collection of surgical outcomes data and expansion of surgical registries in LMICs. Gauge perceptions and attitudes towards surgical outcomes data collection/expansion of surgical registries in LMICs

Intro to role

- Can you briefly explain your role/position?

*Surgeons*

Perceptions/attitudes

- How familiar are you with the concept of surgical outcomes data collection and surgical registries?
- Do you feel data collection and registries are important in the LMIC setting? Why or why not?
- Do you have concerns about surgical registries?
- What benefit do they offer from a surgical standpoint

Barriers/facilitators

- Describe your experience with surgical registries in LMICs
- What do you perceive as the main barriers to surgical registries in the LMIC setting
- Technical – computer software, wifi, IT complexity
- Organizationl 🡪 governance, planning, availability of resources, training supervision, financies, information distribution, promotion of culture
- Behavioral: Data demand, motivation
- What has helped overcome these challenges
- What do you think could overcome these challenges

*Administrators*

Barriers/facilitators

- Have you worked in a hospital that has a surgical registry?
- What were difficulties of having a registry/what would you perceive as difficulties
- What challenges do you foresee in starting a data collection process/registry implementation in a hospital that has never had one (in the LMIC setting)
- How do you foresee/ how have you overcome these challenges
- What resources are necessary to overcome the challenges?

Perceptions/attitudes

- How familiar are you with the concept of surgical outcomes data collection and surgical registries?
- Do you feel data collection and registries are important in the LMIC setting? Why or why not?
- Do you have concerns about surgical registries?
- What benefit do they offer from a surgical standpoint

*Data Collectors*

Perceptions/attitudes

- Describe your feelings towards data collection and surgical registries in the LMIC setting
- Do you feel data collection and registries are important in the LMIC setting? Why or why not?
- Do you have concerns about surgical registries?
- What benefit do they offer
- Did you feel you were making a difference collecting data?

Barriers/facilitators

- Describe your experience collecting data including any challenges
- How did you overcome challenges you faced?
- What challenges persist?
- What could help overcome these challenges?
- If you were to start this process again, how would your approach change?
- Technical – computer software, wifi, IT complexity
- Organizationl 🡪 governance, planning, availability of resources, training supervision, financies, information distribution, promotion of culture
- Behavioral: Data demand, motivation

*Ministry of Health Officials*

Perceptions/attitudes

- How familiar are you with the concept of surgical outcomes data collection and surgical registries? What experience have you had with them?
- Do you feel data collection and registries are important in the LMIC setting? Why or why not?
- How does the government view the value of healthcare data management?

Barriers/facilitators

- Describe your experience with health care data collection/management
- What regulatory and logistical challenges have you seen at the national level?
- What are the challenges from a government standpoint with expanding data collection throughout the country?
- What have been some approaches to overcoming challenges?
- What challenges persist?
