# Supplementary material for: Building better outcomes: A grounded theory approach to understanding creation and management of surgical data systems in Ethiopia
Source: PLOS Glob Public Health. 2026 Jan 30;6(1):e0005355. doi: 10.1371/journal.pgph.0005355 (PMC12857948; doi:10.1371/journal.pgph.0005355)
Supplement: Attachment — Submitted filename: Rebuttal_Letter.pdf [file pgph.0005355.s004.pdf]

WISCONSIN
UNIVERSITY OF WISCONSIN–MADISON

Catherine Kyobutungi PhD

Madhukar Pai Md, PhD, FCAHS

Editors-in-Chief

PLOS Global Public Health

October 6, 2025

Dear Drs. Kyobutungi and Pai

We are excited to resubmit our manuscript entitled "Building better outcomes: A grounded theory approach to understanding creation and management of surgical data systems in Ethiopia." We greatly appreciate the extensive suggestions offered by the reviewers. These insightful recommendations have certainly given us a number of areas to strengthen this manuscript. We feel that all of these recommendations have been thoughtfully considered and either incorporated into the manuscript or acknowledged within this rebuttal letter.

We again thank the editors and reviewers for their time with our work, and look forward to further communication and engagement.

Please find our responses to each point documented below:

Reviewer #1: This is a qualitative study from Ethiopia examining the factors that lead to successful implementation of surgical data registries.

1. There is a typo in the second paragraph of the introduction. The sentence should read "In 2016, Ethiopia was on the first sub-Saharan African countries" - sub-Saharan should be hyphenated and country should be countries.

This has been corrected

2. In the same paragraph, probably District Health Information System should be capitalized.
This has been corrected

3. In the methods, the authors should include a paragraph about how the data registry was set up. What year was it developed? What was the process for setting up the registry? What data is

collected? Who enters the data? Is this person(s) paid for this job? How many people total are involved in maintaining the database? If so, how is it funded? Did the data collectors receive specialized training? On what kind of data system is the data stored? Who has access to the data? Who owns the data? Etc.

We sincerely appreciate the reviewer's interest in our data registry and agree that it is an important component of our broader research efforts. To provide additional context, we have incorporated further details within the introduction. However, we respectfully believe that expanding on this in the methods section of the current manuscript would shift the focus away from the primary objectives of this study. Including extensive information on the registry here risks introducing material that is not directly pertinent and may detract from the clarity of the paper. For readers who are interested in the registry in greater depth, we have provided a citation to the dedicated publication that describes its development and methodology comprehensively.

4. Likewise, it is not clear from the methods and table 1 if all of the people interviewed are directly involved in data collection? Or if you are also seeking opinions from those more peripherally involved.

We have made an addition (underlined) to the paper to further clarify the roles of those interviewed: "A purposive sampling technique was utilized focusing on participants who the research team knew had extensive experience with data systems in some capacity, <u>whether that be through direct data capture, data management, and/or data storage and utilization."</u>

5. The interview guide should be included as a an appendix. Also, was the interview guide vetted with your international partners?

The interview guide was included as a supplemental file named "S1_File"

The interview guide was not formally vetted with our international partners. However, because we employed a laddering interview technique, many of the questions emerged organically, driven by the topics interviewees chose to discuss rather than being predetermined on the guide.

6. In the paper, I would consider removing interview subject labels from specific quotes. In many LMICs, there are limited people in certain roles and by assigning, for example "MOH1" to a certain quote, this person's anonymity may not be protected. I would just keep the quotes but leave the labels out.

We appreciate the reviewer's thoughtful concern and recognize that in many LMIC settings, participants in specific professional roles may be limited in number. However, we feel that we have taken adequate steps to protect anonymity and further feel that including the subject names are exceptionally important for several reasons. First, the labels demonstrate the diversity of perspectives represented and highlight that findings emerged across variety of roles rather isolated voices. Removing identifiers would risk obscuring this heterogeneity, which is critical aspect of qualitative rigor. Second, we acknowledge the possibility of role based recognition, the content of quotations was intentionally kept at a

high-level and focuses more on systemic and structural challenges rather than any type of sensitive or incriminating issues. Finally, providing some degree of attribution, either through role or unique identifier, is standard practice of qualitative research as it enhances the credibility of the data and allows readers to understand how perspectives differ across stakeholder groups.

7. The section that discusses the themes (starting at Theme 1: The resource ecosystem") needs to be written in a more fluid way. It seems very choppy. The quotations are just inserted, without a paragraph explaining the context or the relevance to the overall findings. This section about the themes needs to be completely rewritten to be smoother and tell a story to the reader about what you have found from the research. This is the case for the entire section Themes 1-5. This entire section needs to be rewritten.

We have modified the results section to be more fluid and narrative in nature.

There is also no indication of how many (or what percentage) of interviewees in the study agreed with the sentiment being discussed.

We appreciate the reviewer's suggestion to include how many participants agreed with each sentiment. However, we have grounded our study in qualitative methods that aim to capture depth and meaning rather than quantify agreement. We have utilized qualitative traditions closer to constructivism and interpretivism rather than in a positivist approach, which would focus more on the quantification of agreement. By highlighting a range of perspectives through direct quotations, we are able to show the diversity and richness of views without reducing them to numbers. This approach is consistent with established standards of qualitative rigor and better reflects the aims of our study.

Also, the paragraph that begins Fig 2. types of resources emphasized by role - is this a figure legend? Or is it part of the text?

According to the PLOS Global Public Health submission guidelines: "Figure captions must be inserted in the text of the manuscript, immediately following the paragraph in which the figure is first cited (read order). Do not include captions as part of the figure files themselves or submit them in a separate document." Thus, this is what the "Fig 2. Paragraph" is.

8. The discussion section is a bit unorganized. It should be broken into pieces with subtitles highlighting the different topics, and should also be written more concisely (remove a lot of words).

We have added section headers and streamlined the discussion

9. Figure 1 should be removed. It does not add anything to the paper, as the methods are straightforward and easily understood without the figure.

Figure 1 has been removed

10. The discussion should probably spend more time talking about the framework that was used and how the data can fit into that.

A paragraph has been added that explicitly touches on this point.

11. The conclusion needs to be rewritten. It is a very vague conclusion, which does not explain the main findings of the study. The conclusion should tell the reader, what are the 2-4 things that this study found?

The conclusion has been made more precise and provide the key takeaways from the study.

Reviewer #2: General comment
The manuscript is a valuable and timely contribution to addressing persistent data collection challenges in the surgical space. This thoughtful assessment arrives at a critical juncture in Ethiopia's transition toward digitalization of health care records and identifies critical barriers faced by both surgical data systems and broader healthcare information infrastructure. Appreciation goes to the authors for engaging in such a meaningful and necessary inquiry. Their work offers insight as the country moves toward more efficient data-driven healthcare delivery. However, the paper could benefit from addressing the following comments.

Abstract
There is a mention of interview participant roles twice.

This has been modified such that only one mention is now present in the abstract.

To improve clarity, you might first state the total number of interviews, then list participant types before describing the analysis method. This would streamline the abstract and avoid repetition.

We have shifted to first stating the total number and then the participant types within the methods.

Introduction
In the first sentence of the introduction, the term many countries is vague. It's better to specify which countries, HIC, LMICs? Does focused data here refer to surgical data or any health metrics? I believe the paragraph can be better introduced with better specificity.

We have included that both HIC and LMICs have captured these health metrics, and included a citation. For this statement we do mean any type of health metric.

The authors on the last paragraph of the introduction stated ".. by dissecting the complexity of the situation from different perspectives…". The different perspectives can be specified here to attract the reader's attention … clinicians, data collectors to MOH personnel etc.

This has been added

Methodology
The methods section lacks clarity, unlike the abstract section that states both thematic analysis

and grounded theory were used in combination. I recommend stating this clearly in the methodology section and clarifying and justifying the use of grounded theory in the research.

We have modified the methods section to bring more clarity and also added a sentence explaining why grounded theory was utilized.

The manuscript does not explain how the final sample size of 14 interviews was determined, nor whether thematic saturation was achieved. Including a brief statement on saturation and sampling adequacy would strengthen methodological transparency.

We thank the reviewer for this comment. While the concept of thematic saturation is often discussed in qualitative research, it remains debated and is not universally accepted as a goal or standard. Many qualitative traditions, including ours, do not aim to achieve saturation in the sense of "no new themes emerging," but rather focus on generating sufficient depth and richness of data to meaningfully address the research question. We would like to share a great review article that adequately highlights this debate: Braun, Virginia, and Victoria Clarke. "To saturate or not to saturate? Questioning data saturation as a useful concept for thematic analysis and sample-size rationales." ***Qualitative research in sport, exercise and health*** **13.2 (2021): 201-216.**

Our sample of 14 interviews provided diverse perspectives and adequate data to develop and support the themes presented, aligning with established practices of qualitative rigor. While we agree that more interviews would have potentially strengthened our findings, we ended with 14 for multiple reasons. First, and most plainly, this was the number of individuals who met inclusion criteria and agreed to participate within our recruitment period. Further, because we had to travel the country for these interviews, we were constrained by budget as well as time.

The manuscript also does not mention any intercoder reliability process. Given the interpretive nature of the fifth (latent) theme and the policy implications of the findings, it's important to indicate how the authors maintained rigor in this aspect. If a single person coded all transcripts it's better to mention lack of intercoder reliability in the limitation section.

We thank you for this comment. Quality control and rigor are of utmost importance to us. As there was only one person who coded there was no intercoder reliability. However, we did have a PhD level expert in qualitative methods who oversaw and audited every portion of the project. For coding, the PhD expert and the coder met weekly for approximately 1.5 months to review codes. Further, there was a full code audit of 3 interviews to ensure consistency, reliability and trustworthiness. To ensure transparency, we have included this in the methods section.

Results
Under theme 1, in the first paragraph after the first excerpt, the sentence " The prioritization of each resource was dependent on the role of the interviewer…" appears to have typo error as the role pertains to to the interviewee and not the the interviewer.

This has been edited.

After the third excerpt under theme 1, the paragraph describing a “top down” approach is likely referring to an adaptive mechanism used by MOH for the shortage of resources for training. The authors can be more explicit in their description here for readers' clarity.

This has been elaborated upon.

Theme 3_ In the statement “one of the only hospitals in the country that has EMR,” the use of “only” may unintentionally imply exclusivity, suggesting there is a single such hospital. To enhance clarity and accuracy, it's better to replace it with “one of the few hospitals”, which more precisely conveys the limited, but not singular availability of EMR systems in Ethiopia.

This has been modified.

Theme 4_ The term day laborers working in the chart room can be misleading as it might be referring to janitors, cleaners or guards, who have no role related to data or chart keeping. The phrase in the excerpt from interviewee “They're more of labor workers” might be referring to the inadequacy of their knowledge by comparing them to labor workers. The Authors, however, should be careful in using the term "day laborers" to describe archive staff. This is repeated 3 times under these theme four and should be corrected or the authors should present more substantial evidence of “day laborers” in the chart archive rooms.

We have changed the term day laborer to “archive staff”

Discussion

The discussion section is written in a well elaborated and attractive manner. However, there are several claims that need citation and proper substantiation of claims. Please see the following as an example.

The statement that “financial constraints are a main driver of innovation in LMICs” lacks a supporting citation. Moreover, it appears within an argument that otherwise frames finance as an obstacle. Recommend rephrasing to reflect the paradoxical nature of constraint-driven innovation and including references from frugal innovation literature to strengthen the claim.

We have added a citation as well as elaborated on the paradoxical nature of financial constraints.

On paragraph 4 of the discussion section, the authors mention digitalization as a strategic solution to budget constraints. Several important claims are made about the role of digitalization. These would be stronger if substantiated with concrete examples from literature showing how digital tools simplified complex processes or materials in practice.

We have added specific findings from the literature into this portion of the discussion.

Likewise, the statement that digitalization could “facilitate broader participation in the data acquisition system, in a non-intrusive manner” would benefit from specific instances from

literature or interview excerpts where digital systems demonstrably aided inclusive, low-disruption data collection.

This portion of the discussion has been removed to increase the conciseness of the paper, as suggested by reviewer 1.

The statement referencing a 50% initial capture rate and subsequent deployment of a registry manager provides valuable insight, but it is presented only in the Discussion. The authors should provide a clear context for this discussion point in the result section.

This portion of the discussion has been removed to improve the conciseness of the discussion as recommended by reviewer 1.

The attribution of participant optimism to "Ethiopian culture" is interpretive yet unsupported. To enhance scientific credibility, suggest either framing this as a speculative hypothesis or providing sociocultural references that substantiate this interpretation.

This sentence has been removed

Finally, Interdependent systems theory, the framework developed through infrastructure, people, and budget prioritization is attractive and clearly grounded in the identified themes. However, it may benefit from a deeper articulation of how it innovates beyond existing frameworks like PRISM. The authors could clearly state this as their contribution to existing literature.

A paragraph has been added to address this suggestion.
