# Supplementary material for: Building better outcomes: A grounded theory approach to understanding creation and management of surgical data systems in Ethiopia
Source: PLOS Glob Public Health. 2026 Jan 30;6(1):e0005355. doi: 10.1371/journal.pgph.0005355 (PMC12857948; doi:10.1371/journal.pgph.0005355)
Supplement: Attachment — Submitted filename: Response_to_Reviewers.pdf [file pgph.0005355.s005.pdf]

WISCONSIN
UNIVERSITY OF WISCONSIN–MADISON

Catherine Kyobutungi PhD

Madhukar Pai Md, PhD, FCAHS

Editors-in-Chief

PLOS Global Public Health

January 8, 2026

Dear Drs. Kyobutungi and Pai

We are again excited to submit our manuscript entitled "Building better outcomes: A grounded theory approach to understanding creation and management of surgical data systems in Ethiopia." The continued feedback and recommendations are greatly appreciated, and we feel that the additions have continued to strengthen this work. After careful consideration we feel that each recommendation has been considered and incorporated within this improved manuscript.

We again are greatly appreciative of the time and effort that has been paid to our manuscript from both editors and reviewers, and look forward to further correspondence and eventually featuring this work within your prestigious journal.

Please find our response to each point below:

1. Document clearly that the qualitative interviews were not intended to reach saturation, based on the arguments you present so that your work is not misrepresented or misunderstood.

**We have added the following line within the Methods section (Semi structured interviews subsection): "Interviews were not intended to reach thematic saturation but instead focused on generating sufficient depth and richness of data to meaningfully address the research question at hand."**

2. Because you only dealt with 14 individuals and limited individuals in say the MoH, they can be easily identified using the quote attributions as proxy identifiers. The authors' rebuttal to the reviewer's suggestion is reasonable, however, it might be of interest to the participants not to be identified by those adjacent to the study. Please leave out the attributions with the quotes. This is commonly done with small, niche sample sizes. As a protective measure, if you would like to include them, kindly include consent for interviewees to be represented in this current potentially identifying manner.

**We have removed all quote attributions, and added this statement within the methods section: "Given the small niche sample size, quotations are presented without attribution to protect participant anonymity."**

3. Also kindly include reflections on why the interview guide was not vetted by international partners, as part of reflexivity.

**We appreciate this recommendation and have added the following into our manuscript "The interview guide was not formally vetted by our international partners as it was intended as a flexible framework rather than a fixed instrument, which is consistent with the grounded theory approach. The laddering approach facilitated participants to guide the content and depth of discussion. As such, the majority of the data generation occurred through probing based on participant lead priorities, rather than through strict adherence to pre-specified questions."**

4. Not addressed: "one of the only hospitals in the country that has EMR," the use of "only" may unintentionally imply exclusivity, suggesting there is a single such hospital. To enhance clarity and accuracy, it's better to replace it with "one of the few hospitals", which more precisely conveys the limited, but not singular availability of EMR systems in Ethiopia. Not Addressed

**This has been edited to read "one of the few hospitals in the country"**

5. While the authors note that a code audit was conducted on three interviews and that diverse sampling contributed to the richness of perspectives, these steps do not fully substitute for formal intercoder reliability procedures. Because coding appears to have been conducted primarily by a single researcher, the absence of multiple independent coders should be acknowledged as a methodological limitation, along with its implications for interpretive bias

**This has been added to the limitations section as follows: "Formal intercoder reliability procedures were not employed, as coding was conducted by a single researcher. This represents a potential source of interpretive bias. To enhance rigor and trustworthiness, coding and theme development were conducted under the supervision of a PhD level qualitative methods expert to help decrease the risk of bias or preformed ideas by having iterative analytical meetings and formal coding audits."**
